# Rare Circulating MicroRNAs as Biomarkers of Colorectal Neoplasia

**DOI:** 10.1371/journal.pone.0108668

**Published:** 2014-10-06

**Authors:** Scott V. Adams, Polly A. Newcomb, Andrea N. Burnett-Hartman, Michelle A. Wurscher, Margaret Mandelson, Melissa P. Upton, Lee-Ching Zhu, John D. Potter, Karen W. Makar

**Affiliations:** 1 Public Health Sciences, Fred Hutchinson Cancer Research Center, Seattle, Washington, United States of America; 2 School of Public Health, University of Washington, Seattle, Washington, United States of America; 3 School of Medicine, University of Washington, Seattle, Washington, United States of America; 4 Anatomic Pathology, Group Health Cooperative, Seattle, Washington, United States of America; 5 Centre for Public Health Research, Massey University, Wellington, New Zealand; University of Barcelona, Spain

## Abstract

**Background:**

MicroRNAs (miRNAs) are regulatory RNAs, stable in circulation, and implicated in colorectal cancer (CRC) etiology and progression. Therefore they are promising as early detection biomarkers of colorectal neoplasia. However, many circulating miRNAs are highly expressed in blood cells, and therefore may not be specific to colorectal neoplasia.

**Methods:**

We selected 7 miRNA candidates with previously reported elevated expression in adenoma tissue but low expression in blood cells (“rare” miRNAs), 2 previously proposed as adenoma biomarkers, and 3 implicated in CRC. We conducted a colonoscopy-based case-control study including 48 polyp-free controls, 43 advanced adenomas, 73 non-advanced adenomas, and 8 CRC cases. miRNAs from plasma were quantified by qRT-PCR. Correlations between miRNA expression levels, adjusted for age and sex, were assessed. We used polytomous logistic regression to estimate odds ratios (ORs) and 95% confidence intervals quantifying the association between expression levels of miRNAs and case groups. We also conducted nonparametric receiver operating characteristic (ROC) analyses and estimated area under the curve (AUC).

**Results:**

miRNAs with high expression levels were statistically significantly correlated with one another. No miRNAs were significantly associated with non-advanced or advanced adenomas. Strong (ORs >5) and significant associations with CRC were observed for 6 miRNA candidates, with corresponding AUCs significantly >0.5.

**Conclusions:**

These candidate miRNAs, assayed by qRT-PCR, are probably unsuitable as blood-based adenoma biomarkers. Strong associations between miRNAs and CRC were observed, but primarily with miRNAs highly expressed in blood cells. These results suggest that rare miRNAs will require new detection methods to serve as circulating biomarkers of adenomas.

## Introduction

MicroRNAs (miRNAs) are short, non-coding regulatory RNAs that play critical roles in cell-fate determination and in controlling gene regulation [1]. In addition to regulating expression within cells, cell-derived miRNAs are stable in circulation and have been implicated in the etiology of many cancers including colorectal cancer (CRC) [2]–[4]. Thus, miRNAs are a promising class of potential cancer biomarkers that could augment current CRC screening and surveillance methods [5].

Nonetheless, candidate circulating miRNAs identified for use as potential biomarkers of CRC have also been implicated in the etiology of many cancers as well as other chronic diseases [3], [6], [7]. In addition, most of the previously identified candidate circulating miRNA biomarkers of CRC are also highly expressed in blood cells [8]. This may confound their associations with CRC because small contaminants or variations in blood cell counts could overwhelm disease-specific changes in these circulating miRNAs.

Because current early detection practices can reduce the risk of developing CRC by identifying and removing adenomas, as well as identifying early stage invasive cancers, a potential biomarker to replace or supplement current CRC screening practices must detect premalignant and invasive lesions. Thus, while published studies of circulating miRNAs as CRC biomarkers have generally focused on invasive carcinoma [5], an increasing number of studies have included colorectal adenomas, recognized precursors of CRC [9]–[15].

The objective of this study was to examine whether circulating candidate miRNAs may be useful as screening biomarkers for colorectal neoplasia. To accomplish this objective, we conducted a case-control study of adenomas and colorectal cancers. Our analyses focused on miRNAs previously demonstrated to be overexpressed in adenoma tissue [16], [17], preferentially selecting those of lower abundance in blood cells and plasma of healthy persons [8], or previously proposed as screening markers of adenomas or CRC [9].

## Materials and Methods

### Participant recruitment, study sample, data and biospecimen collection

Participants were part of a clinic-based study of biomarkers of colorectal neoplasia risk conducted at Group Health, a large integrated healthcare delivery system in western Washington State, as described [18], [19]. A total of 738 participants were enrolled in the parent study. Prior to colonoscopy, consenting participants completed a self-administered questionnaire detailing personal and family medical history, as well as demographic, lifestyle, and limited dietary factors. A blood sample was provided for this study at the time of colonoscopy [18], [20]. Blood samples were fractionated and stored at −70°C from collection until accessed for miRNA analysis.

Classification of colorectal polyps biopsied during study colonoscopy has been described [20]. All colorectal polyps received a standardized pathology review by two study pathologists. Adenomas with diameter ≥10 mm, ≥20% villous components, or high grade dysplasia were classified as “advanced.” Carcinomas were identified at endoscopy and received a clinical pathology review.

We excluded a) participants with colorectal lesions or pathology other than adenoma, or carcinoma (e.g., hyperplastic polyps or inflammatory bowel disease); self-reported familial adenomatous polyposis, inflammatory bowel disease, or a previous CRC diagnosis; or age <50 y; b) controls with fewer than 4 remaining plasma samples; and c) cases with no remaining plasma in the repository. With these initial exclusions, the following were eligible for this study: 182 participants with no colorectal polyps or other pathology (controls); 73 with one or more non-advanced adenomas; 43 with one or more advanced adenomas; and 8 with colorectal carcinoma. We randomly sampled 50 controls, frequency matched to the adenoma cases in two age strata, age 50–64 y and >64 y, for our comparison group in the study. Two controls were subsequently excluded because plasma samples were not located in the repository. Thus, final study sample included 48 controls; 73 non-advanced adenoma cases; 43 advanced adenoma cases; and 8 colorectal cancer cases.

### miRNA candidate selection

For this study, we sought to identify candidate miRNAs that could be sensitive and specific biomarkers of colorectal adenoma and carcinoma. We reasoned that circulating levels of a miRNA could be elevated in persons with neoplasia compared to controls if (1) tissue expression was elevated in neoplasia, leading to increased excretion of the miRNA into circulation; or (2) independent of tissue expression level, miRNA excretion was increased in neoplasia. To select candidate miRNAs consistent with condition (1), we relied on published studies of miRNA expression in adenoma tissue [16], [17]. These studies highlighted dozens of miRNAs with expression levels that differed significantly between adenoma and normal tissue. From these we selected miRNAs with higher expression in adenoma tissue than in normal tissue. We did not pursue candidate selection on the basis of condition (2), selectively elevated secretion of miRNAs from colorectal neoplasia, for this study.

Next, we reasoned that because adenomas are relatively small, typically asymptomatic, lesions, the amount of miRNA secreted by adenomas into circulation would likely be small. Therefore observing differences in plasma abundance of candidate miRNAs between adenoma cases and controls would be difficult in the presence of high background plasma levels. Therefore, we cross-referenced candidate miRNAs with the results of Pritchard et al. [8] to eliminate miRNAs candidates above median abundance in plasma, or highly expressed in blood cells. This process resulted in 10 candidate miRNAs (miR-10a, -31, -100, -184, -187-5p, -196a, -203, -224, -660, and -34b-5p). To enable inclusion of miRNA biomarkers previously reported in the literature (next paragraph), we did not include three of these candidates (-224, -660, and -34b-5p).

Finally, we added two miRNAs (miR-29a and -92a) previously reported to be blood-based biomarkers of adenoma [9] and three candidates (miR-17-3p, -125b, -200b) reported to be associated with colorectal cancer etiology or progression [21], [22].

### Isolation of miRNA

miRNAs were extracted from baseline plasma using the Qiagen miRNeasy Serum/Plasma miRNA extraction kit, using adaptations previously described [23]. Briefly, 250 µl of plasma was centrifuged at 3000×g for 7.5 minutes at room temperature (RT). Then, 200 µl of supernatant plasma was transferred to a PhaseLock Gel 15 ml tube (5 Prime Inc.) containing 2.0 mL Qiazol (Qiagen), inverted 10 times, and incubated for 5 minutes at RT. After denaturation (but prior to extraction, described below), each sample received a well-mixed, single-use aliquot containing 30 fmol of synthetic *C. elegans* miRNA (cel-miR-39; Integrated DNA Technologies) and 1 µg of carrier rRNA (Roche Life Sciences, cat# 10 206 938 001) in Qiazol. The sample was inverted 10 times, chloroform (0.2x volume) was added and the sample was inverted again 10 times, and incubated at RT for 2 minutes. The samples were centrifuged at 1500×g for 5 minutes at RT and processed following the remainder of the miRNeasy extraction kit protocol.

### Quantitiation of miRNA

miRNAs were quantified using pre-designed TaqMan MicroRNA assays (Life Technologies Cat#4440887) Following RNA extraction, reverse transcription was completed using the Taq-Man miRNA Reverse Transcription Kit and miRNA-specific stem-loop primers in a 5 µL reaction, again following a previously described protocol [23]. We used a fixed volume (1.67 µL), rather than a fixed mass, of eluted RNA sample and adjusted for differences in RNA recovery by normalization to the synthetic spiked-in cel-miR-39 measured in each sample. We did not use RNA yield to determine RNA input into the reverse transcriptase reaction for two reasons [23]: (1) the yield of RNA from small volumes of plasma is typically to low difficult to accurately quantify, and (2) the 1 µg carrier rRNA added to improve recovery would overwhelm any differences in miRNA mass between samples. In parallel with the experimental samples, we reverse transcribed a 7-point standard curve for each miRNA using synthetic miRNA targets (HPLC-purified, 5′ phosphorylated oligonucleotides, Integrated DNA Technologies) ranging from 3×10^7^ to 7×10^2^ copies.

After reverse transcription, qRT-PCR Taqman assays were performed in triplicate using a commercially available kit (Life Technologies, Thermo Fisher Scientific), scaled down to 5 µL for this application, as described [23]. Assays were run on a 7900 HT Sequence Detection System. Each assay plate contained the standard curve in triplicate, no template controls, external reference plasma controls, positive controls (miRNA from tissue), and inter- and intra-plate duplicates of study samples. Laboratory members were blinded to case-control status and other sample characteristics.

Raw data were analyzed with SDS Absolute Quantification Software version 2.2.3 (Applied Biosystems, Inc.), using the automatic cycle threshold (Ct) setting to assign baseline and threshold for Ct determination. The standard curves for each miRNA were used to calculate miRNA copy number for each miRNA for each sample. Plasma miRNA expression was normalized to a *C. elegans* external spike-in control (*cel*-miR-39) and expressed as normalized copy number, using a median normalization procedure based on the method described [23]. Briefly, TaqMan qRT-PCR assay for cel-miR-39 was run in triplicate, and the mean cel-miR-39 Ct for each sample was calculated and converted into cel-miR-39 copy number based on the cel-miR-39 standard curve. Then, the median value of all the cel-miR-39 copy numbers for all the samples in the study was calculated. For each sample, a normalization factor was calculated by dividing the median value of the cel-miR-39 copy numbers by the cel-miR-39 copy number of the sample of interest. The copy number for the miRNA of interest was then multiplied by the normalization factor to give the normalized copy number.

### Statistical analyses

For miRNAs detected in <50% of plasma samples from controls, we dichotomized miRNA levels as “detected” or “not detected.” For the remaining miRNAs, we used log_10_ (normalized copy number +1) as the independent variable in analyses, referred to as “expression level.”

Using linear regression, we estimated the pairwise partial correlation, independent of participant age (parameterized as a linear continuous variable) and sex, between expression level of miRNAs among controls (N = 48). Correlation was quantified with Pearson correlation coefficients (R). The null hypothesis that R = 0 was tested for each pair of miRNAs (t-test; 44 degrees of freedom). Correlation analyses treated expression level of low-level miRNAs as dichotomized variables (detected/not detected). These analyses were repeated using the continuous expression level for all miRNAs (i.e., without dichotomizing), and “not detected” set to zero.

To examine the association between the expression level of each miRNA transcript and colorectal neoplasia, we used polytomous logistic regression models, adjusting for age (as a linear continuous variable) and sex, to estimate separate odds ratios (ORs) and 95% confidence intervals (CIs) for adenoma, advanced adenoma, and carcinoma. For dichotomized miRNA transcripts, the referent group was comprised of samples with non-detected miRNAs; for more highly expressed miRNAs, ORs were expressed per log-unit (i.e., ten-fold) increase in miRNA expression level. Additional models a) examined adjustment for cigarette smoking (ever/never) and body mass index (as a linear continuous variable); and b) combined non-advanced and advanced adenomas into a single case group.

ROC curves were separately constructed for each miRNA transcript and case group (adenoma, advanced adenoma, carcinoma) compared to controls. The area under the curve (AUC) was estimated with 95% CI using nonparametric methods. miRNA expression levels were parameterized as described for logistic regression.

### Ethics

This observational, human subjects research was reviewed and approved by the Fred Hutchinson Cancer Research Center Institutional Review Board (IR# 4221 and 5839). All participants provided written informed consent.

## Results

Compared to controls, adenoma cases were more often men and overweight or obese (Table 1). The randomly selected controls were more likely to report smoking than adenoma cases.

**Table 1:** Selected characteristics of participants according to colonoscopy results.

			Adenoma					
	Control		Non-advanced		Advanced		Carcinoma	
	(N = 48)		(N = 73)		(N = 43)		(N = 8)	
	N	%	N	%	N	%	N	%
Age (y)								
50–64	21	44	31	42	19	44	4	50
65–79	27	56	42	58	24	56	4	50
Female	28	58	39	53	19	44	3	38
BMI (kg/m^2^)^a^								
<25	19	41	25	34	14	33	4	50
25–29.9	15	33	31	42	19	45	3	38
30+	12	26	17	23	9	21	1	13
Smoking								
never	15	31	31	42	15	35	4	50
former	30	63	37	51	25	58	4	50
current	3	6	5	7	3	7	0	0

a Body mass index.

Six miRNAs (miR-17-3p, -31, -184, -187, -196a, -200b, and -203) were not detected in ≥50% of plasma samples from controls, and were parameterized for analyses as detected/not detected. The expression levels of several miRNAs, primarily those with high expression levels, were strongly correlated (Table 2). Using continuous, rather than dichotomized, expression levels for low-level miRNAs did not materially alter the pattern of correlation (not shown).

**Table 2:** Pairwise Pearson correlation coefficients (R) between miRNAs among controls (N = 48), independent of age and sex.

	miRNA											
miRNA	10a	29a	92a	100	125b	196a^a^	17-3p^a^	31^a^	184^a^	187^a^	200b^a^	203^a^
10a	1											
29a	0.65*	1										
92a	0.49†	0.71*	1									
100	0.48†	0.44‡	0.31§	1								
125b	0.74*	0.71*	0.66*	0.48†	1							
196a^a^	0.11	0.41‡	0.36§	0.23	0.23	1						
17-3p^a^	0.05	0.43‡	0.40‡	0.12	0.20	0.23	1					
31^a^	0.06	0.07	−0.09	0.14	0.04	−0.28	0.08	1				
184^a^	0.08	0.19	0.14	0.06	0.02	0.27	0.07	−0.11	1			
187^a^	0.43‡	0.17	0.28	0.18	0.29	−0.07	−0.13	−0.11	0.30§	1		
200b^a^	0.22	0.48†	0.43‡	0.14	0.22	0.39‡	0.53†	−0.24	0.04	0.06	1	
203^a^	0.02	0.21	0.19	0.04	0.19	0.00	0.39‡	0.09	0.21	−0.06	0.15	1

a Parameterized as detected or not detected.

Test of null hypothesis that R = 0:

*P<0.0001.

† 0.0001<P<0.001.

‡ 0.001<P<0.01.

§ 0.01<P<0.05.

For several miRNA transcripts, expression levels were higher in adenoma and advanced adenoma cases than in controls (Table 3); however, no statistically significant associations between individual miRNA transcripts and either non-advanced or advanced adenomas were observed. In contrast, strong and statistically significant associations were observed between CRC and expression level of miR-10a, -29a, -92a, -100, -125b, and -17-3p. Levels of miR-31, -187, and 203 were below detection in the plasma samples from all of the carcinoma cases.

**Table 3:** Odds ratios (ORs) and 95% confidence intervals (CIs) for the associations of individual miRNA expression levels with colorectal neoplasia.

	Controls		Adenoma								Carcinoma			
			Non-advanced				Advanced							
	Mean	SD	Mean	SD	OR^a^^,^ b	95% CI	Mean	SD	OR^a^^,^ b	95% CI	Mean	SD	OR^a^^,^ b	95% CI
miRNA														
10a	5.9	0.4	6.0	0.4	1.80	(0.71, 4.55)	6.0	0.4	1.71	(0.59, 4.97)	6.4	0.6	13.7	(1.6, 117.8)
29a	5.6	0.4	5.7	0.3	2.05	(0.65, 6.53)	5.7	0.4	2.55	(0.63, 10.3)	6.2	0.7	22.4	(4.0, 125.7)
92a	6.4	0.5	6.4	0.6	1.12	(0.58, 2.18)	6.4	0.5	1.23	(0.61, 2.48)	7.1	0.8	5.9	(1.7, 20.6)
100	3.3	0.9	3.4	0.7	1.19	(0.74, 1.90)	3.3	0.8	1.03	(0.64, 1.67)	4.0	0.7	13.8	(2.7, 70.7)
125b	3.8	0.3	3.9	0.4	1.93	(0.77, 4.83)	3.9	0.4	1.51	(0.47, 4.86)	4.7	0.7	49.6	(4.8, 509.9)
	N	%	N	%	OR^a^^,^ c	95% CI	N	%	OR^a^^,^ c	95% CI	N	%	OR^a^^,^ c	95% CI
196a														
non-detect	25	53	36	49	Ref.		19	44	Ref.		2	25	Ref.	
detect	22	47	37	51	1.17	(0.56, 2.45)	24	56	1.46	(0.63, 3.39)	6	75	3.42	(0.61, 19.22)
17-3p														
non-detect	38	79	56	77	Ref.		27	63	Ref.		3	38	Ref.	
detect	10	21	17	23	1.12	(0.46, 2.74)	16	37	2.16	(0.83, 5.60)	5	63	5.85	(1.26, 27.2)
31														
non-detect	41	85	60	82	Ref.		32	74	Ref.		8	100	—	
detect	7	15	13	18	1.27	(0.45, 3.54)	11	26	2.14	(0.72, 6.34)	0	0		
184														
non-detect	45	94	68	93	Ref.		41	95	Ref.		7	88	Ref.	
detect	3	6	5	7	1.06	(0.24, 4.72)	2	5	0.67	(0.11, 4.18)	1	13	1.85	(0.12, 27.9)
187														
non-detect	45	94	64	88	Ref.		36	86	Ref.		8	100	—	
detect	3	6	9	12	2.02	(0.50, 8.24)	6	14	2.34	(0.54, 10.0)	0	0		
200b														
non-detect	36	75	54	74	Ref.		33	77	Ref.		3	38	Ref.	
detect	12	25	19	26	1.02	(0.44, 2.38)	10	23	0.86	(0.32, 2.32)	5	63	4.64	(0.92, 23.4)
203														
non-detect	44	92	65	89	Ref.		40	93	Ref.		8	100	—	
detect	4	8	8	11	1.32	(0.37, 4.79)	3	7	0.80	(0.16, 3.91)	0	0		

a Compared to controls, adjusted for age and sex.

b OR per log_10_ unit.

c OR comparing detected to non-detected.

Results of ROC analyses to summarize the ability of each miRNA to discriminate between case groups and controls followed the same pattern as the ORs (Table 4). All AUCs for miRNAs discriminating non-advanced or advanced adenoma from controls were not statistically significantly larger than 0.5. For carcinoma, miR-10, -29a, -92a, -100, -125b, and -17-3p each showed AUCs significantly greater than 0.5.

**Table 4:** Area under the curve (AUC) with 95% confidence intervals (CIs) from receiver operating characteristic analysis measuring the ability of miRNA expression levels to discriminate between controls and each case group.

	Non-advanced Adenoma		Advanced Adenoma		Carcinoma	
miRNA	AUC^a^	95% CI	AUC^a^	95% CI	AUC^a^	95% CI
10a	0.58	(0.48,0.68)	0.58	(0.46,0.70)	0.78	(0.58,0.99)
29a	0.57	(0.47,0.67)	0.59	(0.47,0.71)	0.80	(0.64,0.96)
92a	0.54	(0.44,0.63)	0.55	(0.43,0.67)	0.79	(0.62,0.97)
100	0.52	(0.42,0.61)	0.51	(0.39,0.63)	0.80	(0.60,1.0)
125b	0.57	(0.47,0.67)	0.56	(0.44,0.68)	0.89	(0.76,1.0)
196a^b^	0.53	(0.44,0.61)	0.55	(0.44,0.65)	0.64	(0.47,0.82)
17-3p^b^	0.54	(0.47,0.61)	0.58	(0.49,0.68)	0.71	(0.52,0.90)
31^b^	0.53	(0.47,0.59)	0.56	(0.47,0.64)		
18^b^	0.50	(0.46,0.54)	0.49	(0.45,0.54)	0.53	(0.40,0.66)
187^b^	0.53	(0.49,0.58)	0.54	(0.48,0.60)		
200^b^	0.50	(0.43,0.57)	0.49	(0.40,0.58)	0.69	(0.50,0.88)
203^b^	0.51	(0.46,0.55)	0.49	(0.44,0.55)		

a Discriminating from controls.

b AUC based on classification by detection or non-detection.

Combining non-advanced and advanced adenoma into a single case group, or additional adjustment of ORs for participant body mass index or cigarette smoking, did not alter the interpretation of results (not shown).

## Discussion

Our study did not find strong evidence that circulating levels of the selected candidate miRNAs could individually serve as biomarkers for colorectal adenomas. A biomarker with potential clinical importance must have a very strong association (e.g., OR>20) with the outcome of interest for it to discriminate cases and controls with adequate specificity and sensitivity [24]. Results of further analyses of the discriminatory ability of our candidate miRNAs based on ROC curves did not support these miRNAs as biomarkers of adenomas.

We were thus unable to replicate the findings of an earlier report that circulating miR-29a and miR-92a may serve as circulating biomarkers of colorectal adenoma [9], perhaps because of methodologic differences, variability in specimen processing [25], or the substantial expression of miR-29a and miR-92a in blood cells [8]. Notably, Huang *et al.* [9] normalized miRNA levels to endogenous miRNA-16, which is highly expressed in blood, whereas our results are normalized to a spiked-in *c. elegans* miRNA [23].

Our candidate miRNAs were selected prior to the publication of results suggesting miR-21 [11], miR-601 and miR-760 [15], miR-18a [12] and panels of other miRNAs [13], [14] might be biomarkers of colorectal adenoma, and therefore these were not included in our study. Although these earlier studies found evidence that circulating miRNAs could discriminate patients with advanced adenomas from controls, each study proposed different miRNAs despite overlap in miRNA transcripts examined, perhaps suggesting that results are sensitive to uncontrolled specimen preparation and analysis variables. Most of these studies included fewer advanced adenomas than our study [9], [13], [14]; three included a similar number of advanced adenomas as our study [11], [12], [15].

The primary emphasis of our study was on candidate miRNAs with documented differences in expression between adenoma tissue compared to normal tissue [16], [17] but lower expression in blood cells [8] (miR-31, -184, -187, -196a, -200b, and -203). We reasoned that if these miRNAs were detectable in plasma, they would be more specific to colorectal neoplasia, and thus more likely to be useful for screening asymptomatic persons. Our results do not lend much support to these candidate miRNAs as useful for detection of colorectal adenomas using current assay technologies. However, these miRNAs were below the level of detection in more than 50% of plasma samples. This suggests that current qRT-PCR assays lack the sensitivity and precision needed to accurately detect and quantify rare miRNAs in circulation. It is possible that improved technology, such as droplet digital PCR (ddPCR), could allow more accurate assessment of low-level miRNAs and improve biomarker performance. The improved precision of ddPCR over qRT-PCR for measuring circulating miRNAs has been recently demonstrated [26].

Several of our candidate miRNAs (miR-10a, -29a, -92a, -100, -125b, and -17-3p) were strongly associated with invasive CRC, replicating some published reports [5], [9], [10], [12]. Therefore, it is conceivable that these miRNAs, or a panel of miRNAs, could serve as circulating biomarkers of CRC. However, because of the limited number of CRC cases included, and because the study colonoscopies were not all asymptomatic screening exams, our study has limited ability to evaluate miRNAs in association with CRC in the context of screening. Moreover, the clinical utility of such a miRNA-based biomarker for CRC screening is debatable, because current screening technology and recommendations (i.e., endoscopy) target early lesions, and thus can prevent the incidence of invasive CRC. On the other hand, a non-invasive blood test, even one limited to detection of frank carcinoma, may be useful for individuals reluctant to undergo endoscopy. In addition, circulating miRNA levels may be useful in a post-diagnosis setting for disease surveillance.

Our study was limited by a small number of CRC cases and a modest number of adenoma cases, although it was similar in size to previously reported studies of miRNAs and adenomas. We also used plasma samples collected approximately 11 years before miRNA extraction and analysis, which may have influenced our results; although miRNAs were reported to be stable in plasma stored at −80°C and through freeze-thaw cycles [2], any degradation that did occur would have been non-differential and might have biased our results towards the null. Strengths of our study include the well-described population from which the study samples was drawn, the parent study's careful design to evaluate biomarkers and risk factors for colorectal neoplasia [18], [19], and our ability to control for participant characteristics including age, sex, BMI, and cigarette use. Finally, our selection of candidate miRNAs based on two prior reports of miRNA expression in adenoma tissue [16], [17] combined with information on the abundance of miRNAs in circulation and in blood-cells, and previously reports of utility as adenoma biomarkers [9], was a strong and focused rationale for selection, but may have led us to miss other miRNAs.

In summary, we did not observe associations between circulating levels of adenoma-specific, rare candidate miRNAs and the detection of non-advanced or advanced adenoma at colonoscopy; we also did not replicate earlier associations of miR-92a and -29a with adenomas. Strong associations were observed between frank CRC and several miRNAs possibly derived from blood cells [8]. Together, these findings suggest that, although a case-control study may observe an association between a set of miRNAs and CRC, developing a disease-specific blood-based biomarker for asymptomatic persons using circulating miRNAs will be challenging [27], [28]. Future studies would benefit from more sensitive methods to detect rare miRNAs, and careful attention to the specificity of proposed miRNA biomarkers.
